# ROCK2 deprivation leads to the inhibition of tumor growth and metastatic potential in osteosarcoma cells through the modulation of YAP activity

**DOI:** 10.1186/s13046-019-1506-3

**Published:** 2019-12-26

**Authors:** Cinzia Zucchini, Maria Cristina Manara, Camilla Cristalli, Marianna Carrabotta, Sara Greco, Rosa Simona Pinca, Cristina Ferrari, Lorena Landuzzi, Michela Pasello, Pier-Luigi Lollini, Marco Gambarotti, Davide Maria Donati, Katia Scotlandi

**Affiliations:** 1 Department of Experimental, Diagnostic and Specialty Medicine, (DIMES), University of Bologna, Via Massarenti 9, 40126 Bologna, BO Italy; 2 Experimental Oncology Laboratory, IRCCS Istituto Ortopedico Rizzoli, via di Barbiano 1/10, 40136 Bologna, Italy; 3 Department of Pathology, IRCCS Istituto Ortopedico Rizzoli, Bologna, Italy; 4 Clinica Ortopedica III, IRCCS Istituto Ortopedico Rizzoli, Bologna, Italy; 5 Department of DIBINEM, University of Bologna, Bologna, Italy

**Keywords:** Osteosarcoma, ROCK2, YAP, Verteporfin, Metastasis

## Abstract

**Background:**

The treatment of metastatic osteosarcoma (OS) remains a challenge for oncologists, and novel therapeutic strategies are urgently needed. An understanding of the pathways that regulate OS dissemination is required for the design of novel treatment approaches. We recently identified Rho-associated coiled-coil containing protein kinase 2 (ROCK2) as a crucial driver of OS cell migration. In this study, we explored the impact of ROCK2 disruption on the metastatic capabilities of OS cells and analyzed its functional relationship with Yes-associated protein-1 (YAP), the main transcriptional mediator of mechanotransduction signaling.

**Methods:**

The effects of ROCK2 depletion on metastasis were studied in NOD Scid gamma (NSG) mice injected with U-2OS cells in which ROCK2 expression had been stably silenced. Functional studies were performed in vitro in human U-2OS cells and in three novel cell lines derived from patient-derived xenografts (PDXs) by using standard methods to evaluate malignancy parameters and signaling transduction. The nuclear immunostaining of YAP and the evaluation of its downstream targets Cysteine Rich Angiogenic Inducer 6, Connective Tissue Growth Factor and Cyclin D1 by quantitative PCR were performed to analyze YAP activity. The effect of the expression and activity of ROCK2 and YAP on tumor progression was analyzed in 175 OS primary tumors.

**Results:**

The silencing of ROCK2 markedly reduced tumor growth and completely abolished the metastatic ability of U-2OS cells. The depletion of ROCK2, either by pharmacological inhibition or silencing, induced a dose- and time-dependent reduction in the nuclear expression and transcriptional activity of YAP. The nuclear expression of YAP was observed in 80/175 (46%) tumor samples and was significantly correlated with worse patient prognosis and a higher likelihood of metastasis and death. The use of verteporfin, a molecule that specifically inhibits the TEAD–YAP association, remarkably impaired the growth and migration of OS cells in vitro. Moreover to inhibiting YAP activity, our findings indicate that verteporfin also affects the ROCK2 protein and its functions.

**Conclusions:**

We describe the functional connection between ROCK2 and YAP in the regulation of OS cell migration and metastasis formation. These data provide support for the use of verteporfin as a possible therapeutic option to prevent OS cell dissemination.

## Background

Osteosarcoma (OS), a highly aggressive malignant tumor that develops in the bone, preferentially occurs in children and young adults. The prognosis for patients has improved greatly during the past three decades due to the advancement of neoadjuvant and adjuvant chemotherapy in conjunction with surgery, and at present, the 5-year event-free survival rate has reached approximately 70% for patients with localized disease [1–3]. However, the prognosis for metastatic patients remains grim, and the survival rates for patients who present metastases at the time of diagnosis are below 30% [4]. Thus, treating metastatic OS remains a challenge for oncologists, and a deeper understanding of the biology underlying metastasis in OS is an urgent need for the development of novel and more targeted therapeutic options.

The ability of cancer cells to spread to secondary organs outside of the primary tumor site requires mechanical forces exerted via actin cytoskeleton dynamics. The actin status is used as a signaling intermediary by a variety of pathways associated with cancer cell dissemination and metastasis, including the Hippo signaling pathway, an oncosuppressive pathway that plays multiple critical roles in the control of cellular malignancy. Canonical Hippo transduction involves a cascade of serine/threonine kinases that phosphorylate and inhibit Yes-associated protein-1 (YAP) and its coactivator TAZ, promoting their cytoplasmic retention and/or subsequent degradation. When Hippo signaling is ‘off’, YAP and TAZ translocate to the nucleus, where they interact with the transcription factors TEAD1–4 to induce the expression of target genes responsible for cellular proliferation, differentiation and survival [5]. The dysregulation of Hippo signaling and/or YAP activity occurs frequent in a variety of human cancers [6], including OS, as YAP is highly expressed in both human and mouse OS. YAP suppression sharply decreases cell proliferation, cancer stemness and tumorigenicity [7–9], thereby acting as a potential therapeutic target for tumors. In addition to operating in Hippo signaling, YAP also senses and mediates the integrity of the actomyosin cytoskeleton and the intracellular mechanotransduction pathway [10–13]. The actin status is also controlled by the Rho/Rho-associated coiled-coil containing protein kinase (ROCK) pathway, which can sustain and promote YAP activity through the phosphorylation of several molecular targets that are induced by Rho-associated coiled-coil containing protein kinase 1 and 2 (ROCK1 and ROCK2) [11, 14]. Thus, the Hippo pathway, the cytoskeleton, Rho/ROCK and YAP/TAZ may form a complex molecular network of multilayered interactions with feedback mechanisms, whose connections are still poorly understood and may differ in diverse cellular contexts. In OS, we have previously highlighted the importance of ROCK2, rather than ROCK1, as a crucial mediator of cell migration and invasion [15]. In this study, we analyzed the impact of ROCK2 depletion on OS metastasis and its functional connections with YAP activity. We also tested verteporfin, a small molecule that specifically inhibits the TEAD–YAP association [16], as a potential therapeutic agent for OS.

## Methods

### Cell lines

The U-2OS OS cell line was obtained from the American Type Culture Collection (ATCC). The primary cultures PDX-OS#2-C1, PDX-OS#16-C2 and PDX-OS#25-C1 were recently obtained from OS patient-derived xenografts (PDXs) after one or two passages in animals [17]. Patient informed consent was obtained for the establishment of the PDX models**.** All cell lines were tested for mycoplasma contamination (Mycoalert Mycoplasma Detection Kit, Lonza) before use. Cell lines were immediately expanded to generate liquid nitrogen stocks and were never passaged for more than 1 month after thawing. Cells were grown in Iscove’s modified Dulbecco’s medium (IMDM) supplemented with 10% inactivated fetal bovine serum (FBS) (Euroclone), 100 units/ml penicillin and 100 μg/ml streptomycin (Sigma). Cells were maintained at 37 °C in a humidified 5% CO_2_ atmosphere.

### Stable silencing

For stable silencing, a short hairpin RNA (shRNA) plasmid (pSilencer 2.1-U6 Neo vector; Ambion) expressing ROCK2 siRNA (Fw: 5′-GATCCCGGCAACTGGCTCGTTCAATTTTCAAGAGA TTAACTTGCTCGGTCAACGTTTTTTGGAA-3′; Rw: 5′-AGCTTTTCCAAAAAACGTTGACCGAGCAAGTTAATCTCTTGAAAATTGAACGAGCCAGTTGCCGG-3′) was created, and U-2OS cells were transfected using the calcium phosphate transfection method (Life Technologies). Stable transfectants expressing shRNA-ROCK2 (U-2/shROCK2#78 and #46) or nontargeting shRNA sequences (U-2/SCR pool) were obtained after selection in neomycin (500 μg/ml) (Sigma).

### Treatments

For transient ROCK2 silencing, cells were transfected with small interfering RNA (siRNA) sequences targeting ROCK2 (ON-TARGETplus SMARTpool, Human ROCK2, Dharmacon) or irrelevant targets (ON-TARGETplus Non-targeting siRNA). For ROCK2 inhibition, the ROCK2 inhibitor N-(2-(2-(dimethylamino)ethoxy)-4-(1H-pyrazol-4-yl)phenyl)-2,3dihydrobenzo[b]1, 4 dioxine-2-carboxamide (Stemolecule ROCK2 Inhibitor, Stemgent) was used. To inhibit YAP activity, tests were performed with the YAP inhibitor verteporfin (Sigma). Both compounds were dissolved in dimethyl sulfoxide (DMSO; Sigma-Aldrich). Working solutions were prepared in IMDM immediately before use.

### Motility assay

Cells (1 × 10^5^) were pretreated with or without the YAP inhibitor verteporfin (2 μM) for 24 h, after which they were analyzed for their migration ability. A motility assay was performed using Transwell chambers (Costar) with 8-μm pore size polyvinylpyrrolidone-free polycarbonate filters (Nucleopore). Cells were seeded in IMDM with 10% FBS in the upper compartment and were incubated for 18 h at 37 °C. The number of cells that migrated toward the filter to reach the lower chamber was counted after fixation with methanol and staining with Giemsa (Sigma).

### Wound-healing assay

A total of 2 × 10^5^ U-2OS cells were seeded in 60-mm Petri-dish well plates. Cells were allowed to grow to 100% confluence. The cell monolayer was scraped in a straight line to create a scratch with a p200 pipet tip. The debris was removed, and the medium was replaced with IMDM with 10% FBS with or without 2 μM verteporfin. Cells were kept in a tissue culture incubator at 37 °C, and pictures were taken at 0, 3 and 6 h.

### Cell growth inhibition

To perform cell culture experiments, OS cells (2X10^5^/well for U-2OS or 4 × 10^5^/well for PDX-OS primary cultures) were plated, and verteporfin (0.1–10 μM) was added after 24 h. Cells were exposed to the drug for up to 96 h before being counted by Trypan blue vital dye exclusion (Sigma). In parallel, cells were treated with DMSO-containing medium as a control. The highest final concentration of DMSO in the medium was < 0.3%, and DMSO had no effect on cell growth.

Anchorage-independent growth was measured in 0.33% agarose (Sea-Plaque; Lonza) with a 0.5% agarose underlay. OS cells (10,000 for U-2OS or 100,000 for PDX-OS#16-C2) were plated in semisolid medium with or without verteporfin (2 μM) and were incubated at 37 °C in a humidified 5% CO_2_ atmosphere. Colonies were counted after 10 and 14 days for U-2OS or PDX-OS#16-C2, respectively.

### Immunofluorescence

Cells grown on coverslips were treated with verteporfin as described above. Cells were fixed in 4% paraformaldehyde were permeabilized with 0.15% Triton X-100 (Sigma) in phosphate-buffered saline or in methanol and were incubated with the following antibodies: anti-YAP (sc-271134, dilution 1:25), anti-β-catenin (sc-7963, dilution 1:50), and anti-ROCK2 (sc-398,519, dilution 1:50) that were all purchased from Santa Cruz Biotechnologies; and anti-N-cadherin (BD Transduction Labs, 610921, dilution 1:100). Anti-mouse FITC (Thermo Scientific, #31569, dilution 1:100) or anti-goat IgG NL493 (FITC equivalent R&D, #NL003, dilution 1:50) were used as secondary antibodies. Nuclei were counterstained with Hoechst 33256 (Sigma). Images were acquired using a Nikon ECLIPSE 90i microscope and were then analyzed with NIS-Elements software (Nikon).

### In vivo experiments

Female, 5 weeks old, immunodeficient NOD Scid gamma (NSG) mice were obtained from Charles River, Italy. Groups of 6 mice received injections of 10^7^ U-2OS cells subcutaneously. Tumor growth was measured weekly and tumor volumes were calculated as π/2·[√(a·b)]^3^/6, where a and b are the two maximal diameters. After 9–10 weeks, animals were sacrificed by CO_2_ inhalation and cervical dislocation, and an accurate necropsy was performed. Tumors were removed for further studies; lungs were perfused with black India ink and fixed. Lung metastases were then counted under a dissecting microscope.

### RNA extraction and qPCR

Total RNA from snap-frozen tissue samples and cell lines was isolated using TRIzol Reagent (Thermo Fisher Scientific - Life Technologies). RNA quality and quantity were assessed by NanoDrop analysis (NanoDrop ND1000, Thermo Scientific) and by electrophoresis. Total RNA from each sample was reverse transcribed into complementary DNA (cDNA) using a High-Capacity cDNA Reverse Transcription Kit (Thermo Scientific - Applied Biosystems, #4368814) according to the manufacturer’s protocols. Quantitative PCR (qPCR) was performed on a ViiA7 system (Life Technologies) using TaqMan Universal PCR Master Mix (Thermo Fisher Scientific - Applied Biosystems, #4304437) and SYBR Green PCR Master Mix (Thermo Fisher Scientific - Applied Biosystems, #4312704). Predesigned TaqMan probes (Thermo Fisher Scientific - Applied Biosystems) were used for Connective Tissue Growth Factor (*CTGF*) (Hs00170014) Cysteine Rich Angiogenic Inducer 61 (*CYR61*) (Hs00155479) and Cyclin D1 (CCND1) (Hs00765553). The primers used are *ROCK2* forward 5′- CAACTGTGAGGCTTGTATGAAG-3′ and reverse 5′-TGCAAGGTGCTATAATCTCCTC-3′; GAPDH forward: 5′-GAAGGTGAAGGTCGGAGTC-3′, reverse: 5′-GAAGATGGTGATGGGATTTC-3′.Relative quantification was performed in tumor samples with the ΔCT method (relative abundance, RA = 2^- ΔCT^) while the ΔΔCT method (relative quantification, RQ = 2^- ΔΔCT^) was used for cell line analysis. The expression levels of the target genes were normalized to those of the housekeeping gene *GAPDH* (Hs99999905_m1). Untreated cells (CTRL) or cells exposed to an shRNA against irrelevant targets (SCR) were used as controls.

### Western blotting

Subconfluent cells were treated as described above and were processed for Western blotting following standard procedures, using total protein lysates or fractionated proteins, where appropriate. Cytoplasmic proteins were obtained using the lysis buffer containing 50 mmol/L HEPES (pH 7.5), 150 mmol/L NaCl, 1% Triton X-100, 1.5 mmol/L MgCl2, EGTA, 10 mmol/L (pH 7.5), glycerol 10%, and inhibitors (0.1 mmol/L Na3VO4, 1% phenylmethylsulfonyl fluoride, and 20 mg/mL aprotinin). After the collection of cytoplasmic proteins, the nuclei were lysed with the nuclear buffer containing 20 mmol/L HEPES (pH 8), 0.1 mmol/L EDTA, 5 mmol/L MgCl2, 0.5 mol/L NaCl, 20% glycerol, 1% Nonidet P40, and inhibitors (as above). The following primary antibodies were used: anti-ROCK2 (Abcam, #ab125025, dilution 1:12000); anti-YAP (Cell Signaling, #14074, dilution 1:1000) anti-GAPDH (Santa Cruz, sc-25,778, dilution 1:5000) and anti-Lamin B (Santa Cruz, sc-6216, dilution 1:5000). Anti-rabbit (GE Healthcare, #NA934), anti-mouse (GE Healthcare, #NA931) or anti-goat (Santa Cruz, sc-2020) secondary antibodies conjugated to horseradish peroxidase were used, and bands were visualized with enhanced chemiluminescence Western blotting detection reagents (EuroClone).

### Patients

Patients with localized primary OS who were enrolled in prospective studies and were treated at the Rizzoli Institute were included in the current analysis. The present study included 175 tumor samples from biopsy specimens (obtained before chemotherapy and preserved in archival paraffin-embedded tissue blocks) that were available for immunohistochemical analysis and had adequate tissue. All tumors were classified as stage II conventional high-grade OS [18]. Chemotherapy was given before and after surgery. Chemotherapy protocols based on doxorubicin, high-dose methotrexate, cisplatin and/or ifosfamide have been previously described [19–22]. The surgical procedures took into account the location and extent of the tumor and the life expectancy of the patient. A limb-salvage procedure was performed in 158 patients (90%). The surgical margins of the tumor specimens were histologically defined according to the system of Enneking [18]. The extent of tumor necrosis was evaluated with a previously described semiquantitative method [23]. Adverse events were defined as a recurrence of the tumor at any site (local or systemic) or death during remission. Relapse-free survival (RFS) was calculated from the date of the initial diagnosis. The median follow-up of the population was 95 months (range 2–415 months). Clinical and follow-up data were updated until December 2018. The rates of RFS and overall survival (OVS) were 51.4 and 69.7%, respectively. Table 1 summarizes the clinical and pathological characteristics of the 175 patients.

**Table 1:** Clinicopathological features of OS patients evaluated for YAP expression by immunohistochemistry (IHC) in 175 tissue samples

Characteristics	N	%	Association with prognosis (RFS)^a^	Association with survival (OVS)^b^
Gender			*P* = 0.67	*P* = 0.92
Female	72	41.1		
Male	103	58.9		
Age			*P* = 0.37	*P* = 0.10
≤ 14 years	82	46.9		
> 14 years	93	53.1		
Surgery			*P* = 0.47	*P* = 0.91
Resection	158	90.3		
Amputation	12	6.9		
Rotation plastic	5	2.8		
Margins			*P* = 0.10	*P* = 0.36
Adequate	147	84.0		
Inadequate	28	16.0		
Chemotherapy response (Necrosis)			*P* = 0.05	*P* = 0.21
Good	105	60.0		
Poor	69	39.4		
NA	1	0.6		
RFS (status)				
NED	90	51.4		
REL	85	48.6		
OVS (status)				
Living	122	69.7		
Dead	53	30.3		

Associations with prognosis were calculated by univariate analysis using the log-rank test

*OS* osteosarcoma, *NED* no evidence of disease, *REL* relapsed, *RFS* relapse-free survival, *OVS* overall survival

^a^RFS (median follow-up: 95 months; range 2–415 months)

^b^OVS (median follow-up: 156 months; range 7–415 months)

### Immunohistochemistry

An avidin–biotin–peroxidase procedure was used for immunostaining (Vector Laboratories). Antigen retrieval was performed using citrate buffer (pH 6.0), followed by incubation with anti-YAP (sc-271134, dilution 1:50) or anti-ROCK2 (sc-398,519, dilution 1:50). In human tumor samples, we used a semiquantitative score for YAP immunostaining to evaluate its level of expression together with an analysis of its intracellular location to evaluate its activity. Patients were classified as positive when the nuclear positivity of YAP was detected. The expression levels were scored as follows: negative, when no staining was observed; positive, including weak (+ − -), moderate (++−), and strong (+++) positivity levels.

### Statistical analysis

Differences among the means were analyzed using Student’s t tests. For analysis of incidence and median number of lung metastasis Fisher’s exact test and Wilcoxon’s rank sum test were used. CalcuSyn2 software (Biosoft) was used to calculate the IC_50_ values. The association between YAP expression and RFS or OVS was estimated by Cox proportional hazards regression analysis. RFS and OVS were plotted using the Kaplan-Meier method, while the log-rank test was used to calculate the univariate statistical significance of the observed differences. RFS was calculated as the time from diagnosis to the occurrence of adverse events, which were defined as recurrence or metastases at any site. OVS was defined as the time from diagnosis to cancer-related death. Survivors or patients who were lost to follow-up were censored at the last contact date. All factors that were significantly associated with RFS in the univariate analysis were entered into a Cox proportional hazards model for multivariate analysis. Values for the 95% confidence interval (CI) of the hazard ratios (HRs) are provided [24]. The Chi square test was used for association data. Statistical analyses were performed with SPSS software, version 22.0.

## Results

### ROCK2 deprivation inhibits the in vivo growth and metastatic ability of OS cells through the modulation of YAP activity

**Fig. 1:**
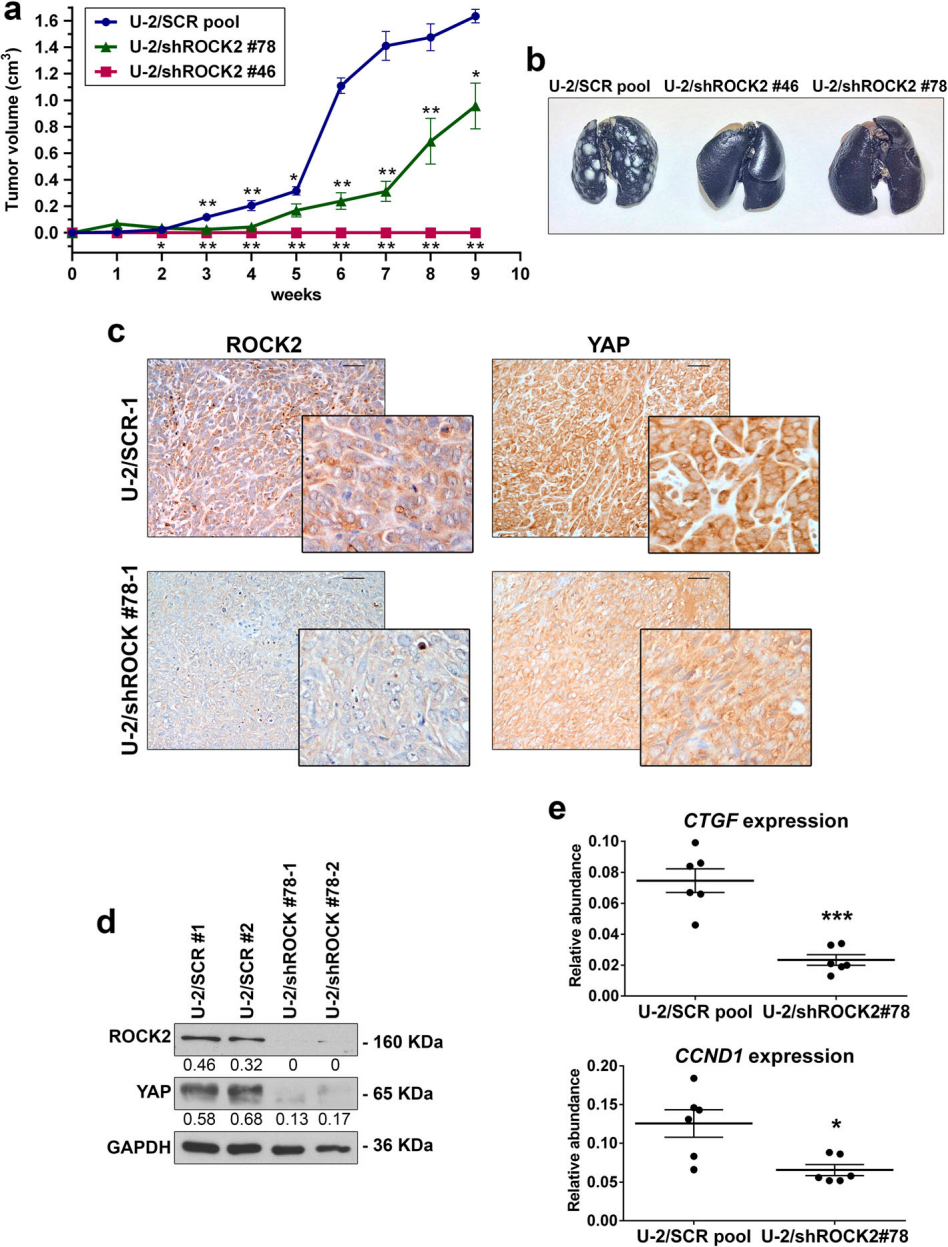
Silencing ROCK2 in OS cells impairs tumor growth and metastasis in mice and downregulates YAP expression and activity. **a** Tumor volume and **b** representative images of lung metastases of mice injected subcutaneously with U-2OS cells modified for ROCK2 expression. *n* = 6 animals per group. *p < 0.05, **p < 0.001 vs. control (U-2/SCR pool) by the Student’s *t* test. **c** ROCK2 and YAP expression were evaluated by the immunostaining of paraffin-embedded tissue samples. Representative images from one tumor developed after injection of control (U-2/SCR) or silenced cells (U-2/shROCK2#78). Scale bar, 50 μm; 200x of magnification with zoomed insert to show details. **d** Western blotting of snap-frozen tissue samples from two representative tumors that formed in mice injected with control (U-2/SCR) or silenced cells (U-2/shROCK2#78). GAPDH was used as a loading control. ROCK2 or YAP signals were quantified against GAPDH and reported as ratio of adjusted volume optical density (OD/mm^2^). **e** YAP activity was evaluated by measuring the relative mRNA expression of its downstream targets *CTGF* and *CCND1* by qPCR. Scatter plot analysis of their expression in the control and U-2shROCK2#78-derived xenografts (*n* = 6) is shown. The 2^−ΔCT^ method, where ΔCT = CT target gene – CT GAPDH, was used. Bars represent mean ± SE, *p < 0.05, ***p < 0.001, Student’s t test

**Fig. 2:**
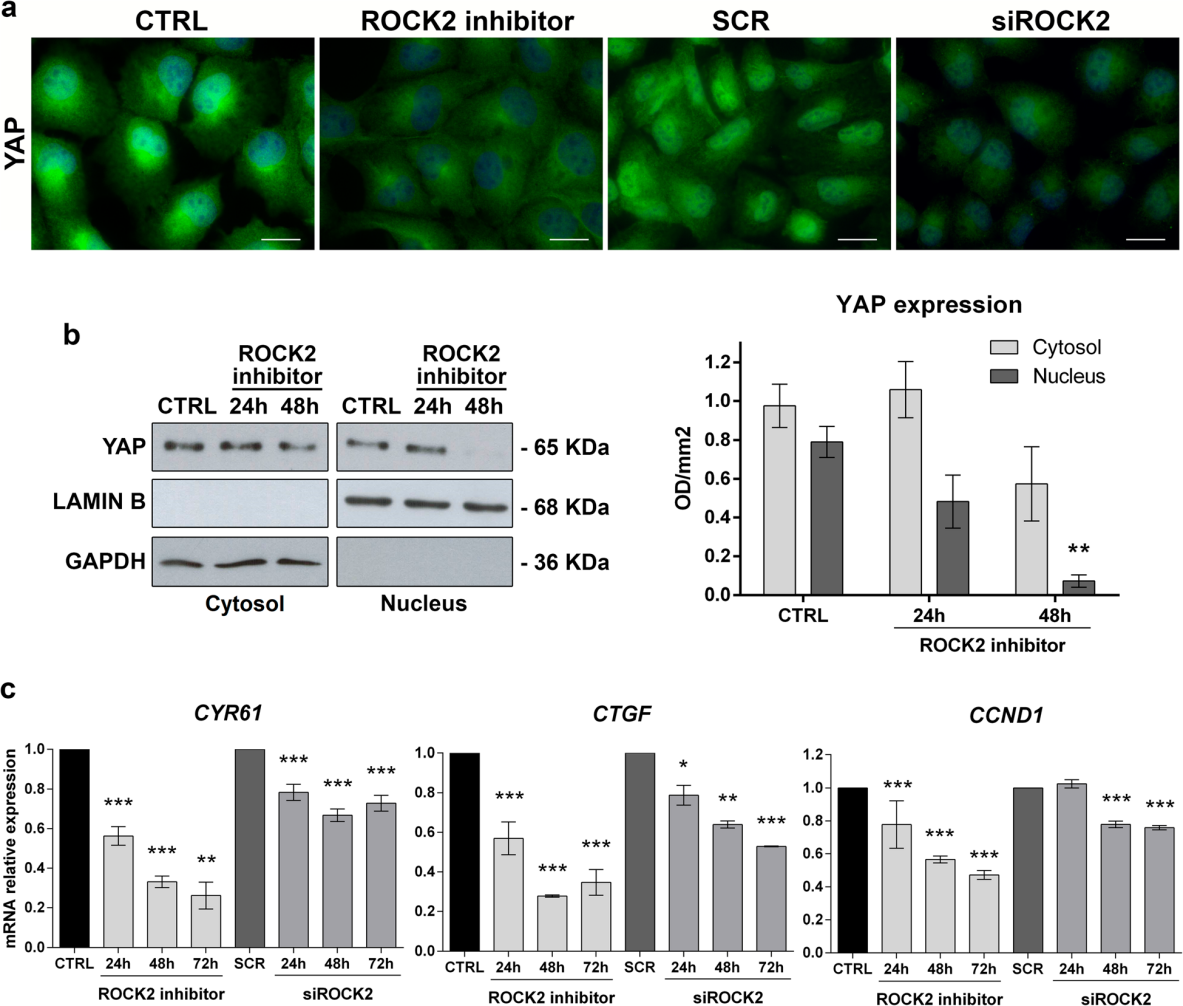
ROCK2 silencing reduces YAP expression and YAP-mediated transcriptional activity. **a** Immunofluorescence staining of YAP in U-2OS cells after 24 h of exposure to the Stemolecule™ ROCK2 Inhibitor (10 μM) or siRNA sequences targeting ROCK2 (siROCK2) or irrelevant target sequences (SCR). Digital images were taken under identical conditions using the image analysis software NIS-Elements (Nikon Italia); scale bar, 20 μm. **b** Western blotting of YAP in cytoplasmic and nuclear fractions of U-2OS cells after 24–48 h of exposure to ROCK2 inhibitor together with densitometric analysis. YAP signal was quantified against GAPDH or LAMIN B and reported as ratio of adjusted volume optical density (OD/mm^2^). Data are presented as the mean ± standard error (SE) of three separate experiments (** *p* < 0.01, Student’s t test) **c** qPCR analysis of the expression of *CYR61*, *CTGF* and *CCND1* in U-2OS parental cells after 24-h to 72-h treatments. Data are shown as 2-^ΔΔ*Ct*^. GAPDH was used as a housekeeping gene. Data are presented as the mean ± standard error (SE) of three separate experiments (* *p* < 0.05, ** *p* < 0.01, *** *p* < 0.001, Student’s t test)

The stable silencing of ROCK2 was induced in U-2OS cells to evaluate the impact of this kinase on the metastatic capability of OS cells. Cells transfected with nontargeting sh-sequences were used as controls. The depletion of ROCK2 markedly reduced tumor growth when tumor cells were injected into immunodeficient NSG mice (Fig. 1a; Table 2) and completely abolished the pulmonary metastatic potential of these cells (Fig. 1b; Table 2). According to our previous observations, ROCK2-silenced OS cells (namely, U-2/shROCK2#46 and U-2/shROCK2#78) showed increases in the expression and cell-membrane recruitment of N-cadherin and β-catenin, a weak migration capability in Transwell chambers, and a weak ability to form colonies in anchorage-independent conditions (Additional file 1: Figure S1). In U-2/shROCK2 tumors, YAP expression was remarkably decreased (Fig. 1c, d and Additional file 2: Figure S2), and its activity was inhibited, as indicated by a decrease in the expression of the YAP/TEAD-regulated genes *CTGF* and *CCND1*(Fig. 1e). Consistently, we observed a remarkable reduction in the expression and activity of YAP either when ROCK2 was inhibited by the specific Stemolecule ROCK2 inhibitor [15] or by the transient exposure of cells to siRNA sequences. In addition to the immunofluorescence analysis (Fig. 2a), biochemical fractionation of nuclear and cytoplasmic fractions after ROCK2 inhibition clearly showed the time-dependent nuclear abrogation of YAP expression (Fig. 2b). Accordingly, the expression of the YAP-TEAD-regulated genes *CTGF*, *CYR61* and *CCND1* was found to be significantly inhibited when ROCK2 activity is hampered (Fig. 2c), confirming the functional association between ROCK2 and YAP activity. The inhibition of the YAP targets was maintained for at least until 72 h after the cell treatments.

**Table 2:** Tumorigenicity and metastatic ability of U-2OS cells after the depletion of ROCK2

Cells	Tumor			Lung metastases		
	Incidence	Latency (mean days ± SEM)	Volume at 9 weeks (mean cm^3^ ± SEM)	Incidence	Median number	Individual values
U-2/SCR pool	6/6	23.2 ± 1.2	1.635 ± 0.051	6/6	> 200	> 200, > 200, > 200, > 200, > 200, > 200
U-2/sh ROCK2 #78	6/6	37 ± 2.9	0.958 ± 0.233^*^	0/6†	0‡	0, 0, 0, 0, 0, 0
U-2/sh ROCK2 #46	0/6	NA	0^**^	0/6†	0‡	0, 0, 0, 0, 0, 0

†*p* = 0.01, Fisher’s exact test, vs U-2/SCR pool

^*^p < 0.05, ^**^p <0.0001, Student’s t test, vs. U-2/SCR pool

‡*p* < 0.01, Wilcoxon’s rank sum test, vs. U-2/SCR pool

### YAP activation is associated with a worse prognosis for OS patients

**Fig. 3:**
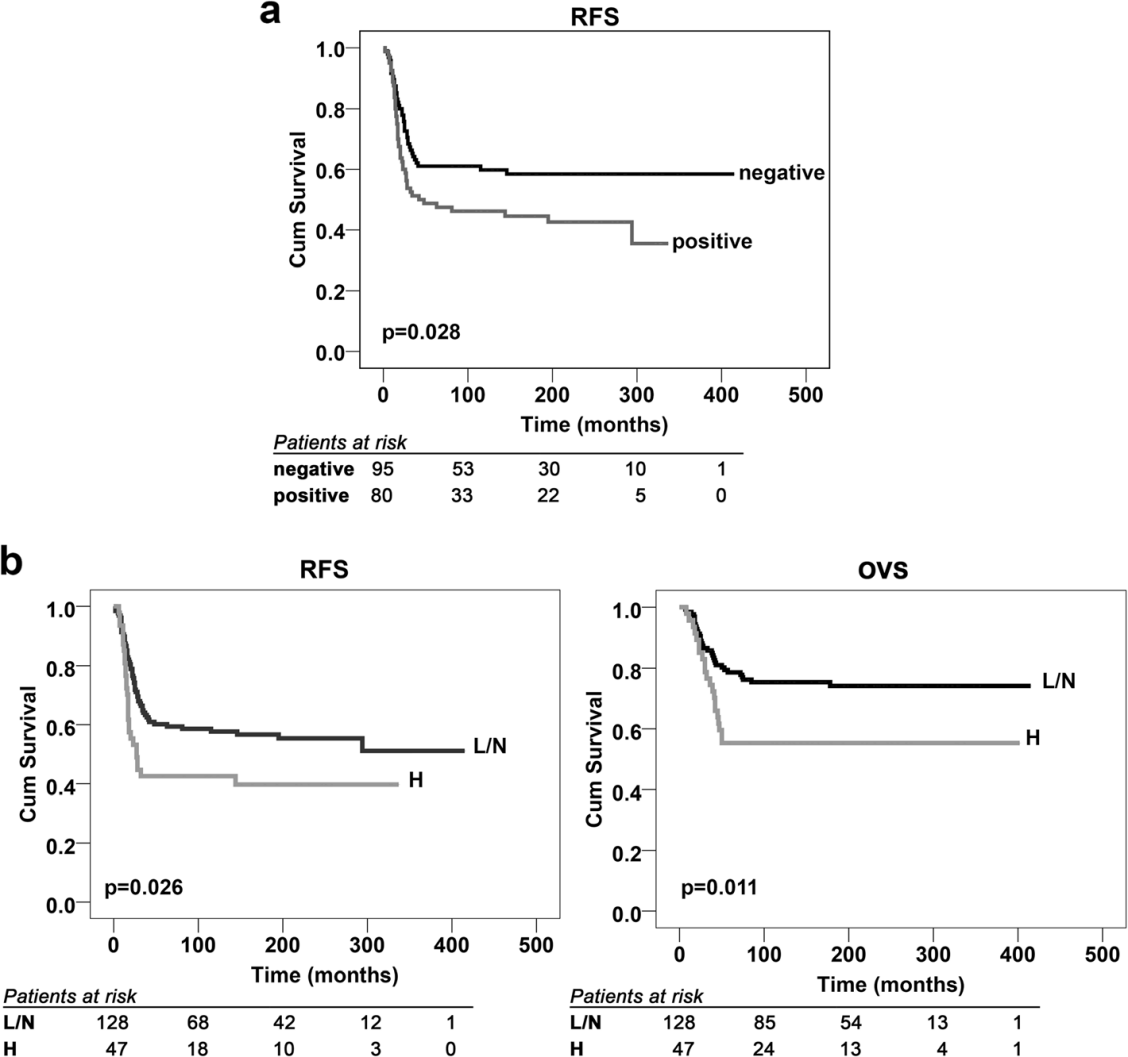
YAP nuclear expression correlates with poor outcome in OS patients. **a** Prognostic impact of the presence (positive) or absence (negative) of YAP in the nucleus of OS cells. **b** Prognostic impact of high (H) versus low or no (L/N) YAP expression in the nucleus. RFS and/or OVS were evaluated based on the Kaplan-Meier curves and log-rank test in 175 cases that were analyzed by immunostaining. The time scale refers to the months from diagnosis. The number of patients at risk in positive or negative as well as in H and L/N samples is listed below each time interval

Positivity for YAP immunostaining, either at the cytoplasmic or nuclear level, was detected in the majority of primary OS (131/175; 75%), while the nuclear localization of the protein, which is directly related to its activity [5], was found in 80 out of 175 patients (46%). Adverse metastatic events occurred in 42 of the 80 (53%) patients with nuclear YAP expression and in 34 of the 95 (36%) patients with inactive YAP (*P* = 0.026, Chi square test). Accordingly, Kaplan-Meier curves (Fig. 3a) confirmed that the presence of YAP in the nucleus of OS cells was significantly associated with a decreased probability of remaining event-free after diagnosis (*P* = 0.028, log-rank test). Cox multivariate regression analysis was performed for the variables that were found to be associated with RFS by univariate analysis and showed that the nuclear status of YAP was the only independent risk factor for poor outcomes (Table 3). To further confirm this observation, we used the strong expression of YAP in the nucleus (++/− and +++) to stratify patients as high-expressors (H) or low-expressors/nonexpressors (L/N) (47 vs 128 patients). Kaplan-Meier curves confirmed that very high YAP expression in the nucleus significantly affected both RFS and OVS in OS patients (Fig. 3b), indicating that the level of YAP activity is critical for patient outcomes. Consistently, the percentage of patients who died from this disease was significantly higher in those with high levels of active YAP (dead patients: 21/47, 45% vs 32/128, 25%, respectively; *p* = 0.012, Chi square test).

**Table 3:** HR of relapse for variables associated with RFS by univariate analysis in 175 patients (estimated by Cox proportional hazards regression multivariate analysis)

Variables associated with worse RFS	HR	95% CI	*P* value
Response to chemotherapy: poor	1.222	0.987–1.513	0.066
YAP localization: nuclear	1.620	1.056–2.483	0.027

### Targeting YAP with verteporfin inhibits the malignancy of OS cells

**Fig. 4:**
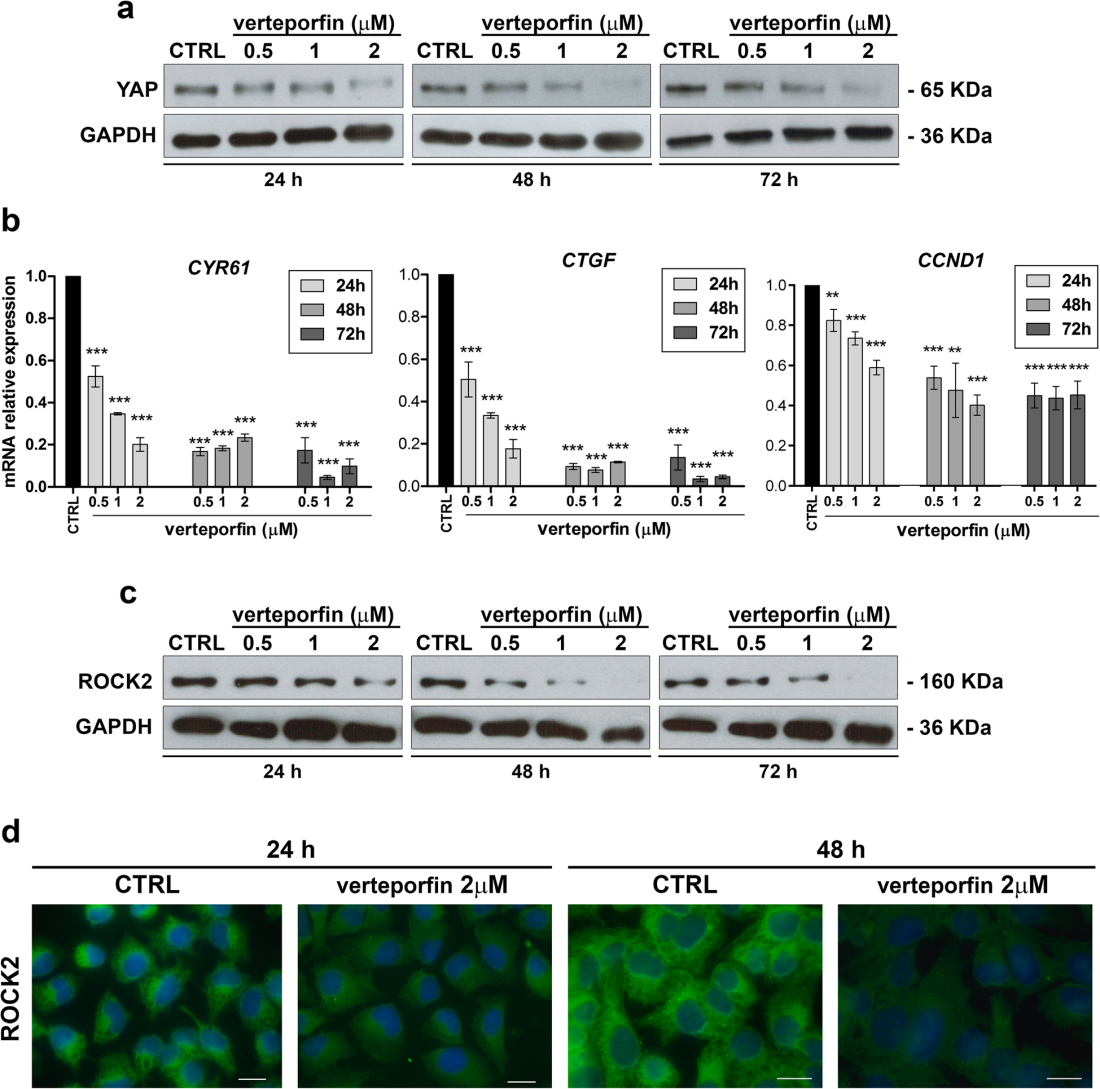
Verteporfin inhibits YAP expression and activity as well as ROCK2 expression. **a** Western blot analysis of YAP in untreated (CTRL) or verteporfin-treated U-2OS cells. GAPDH was used as a loading control. **b** Dose- and time-dependent qPCR analysis of the expression of *CYR6*1, *CTGF* and *CCND1*, conventional YAP downstream targets, in U-2OS parental cells. Data are shown as 2-^ΔΔ*Ct*^. Data are presented as the mean ± SE of three separate experiments (* *p* < 0.05, ** *p* < 0.01, *** *p* < 0.001, Student’s t test). GAPDH was used as a housekeeping gene. **c** Western blot analysis of ROCK2 in untreated (CTRL) or verteporfin-treated U-2OS cells. GAPDH was used as a loading control**. d** Expression of ROCK2 by immunofluorescence in U-2OS cells treated with verteporfin for 24–48 h. Digital images were taken under identical conditions using the image analysis software NIS-Elements (Nikon Italia). Scale bar, 20 μm

**Fig. 5:**
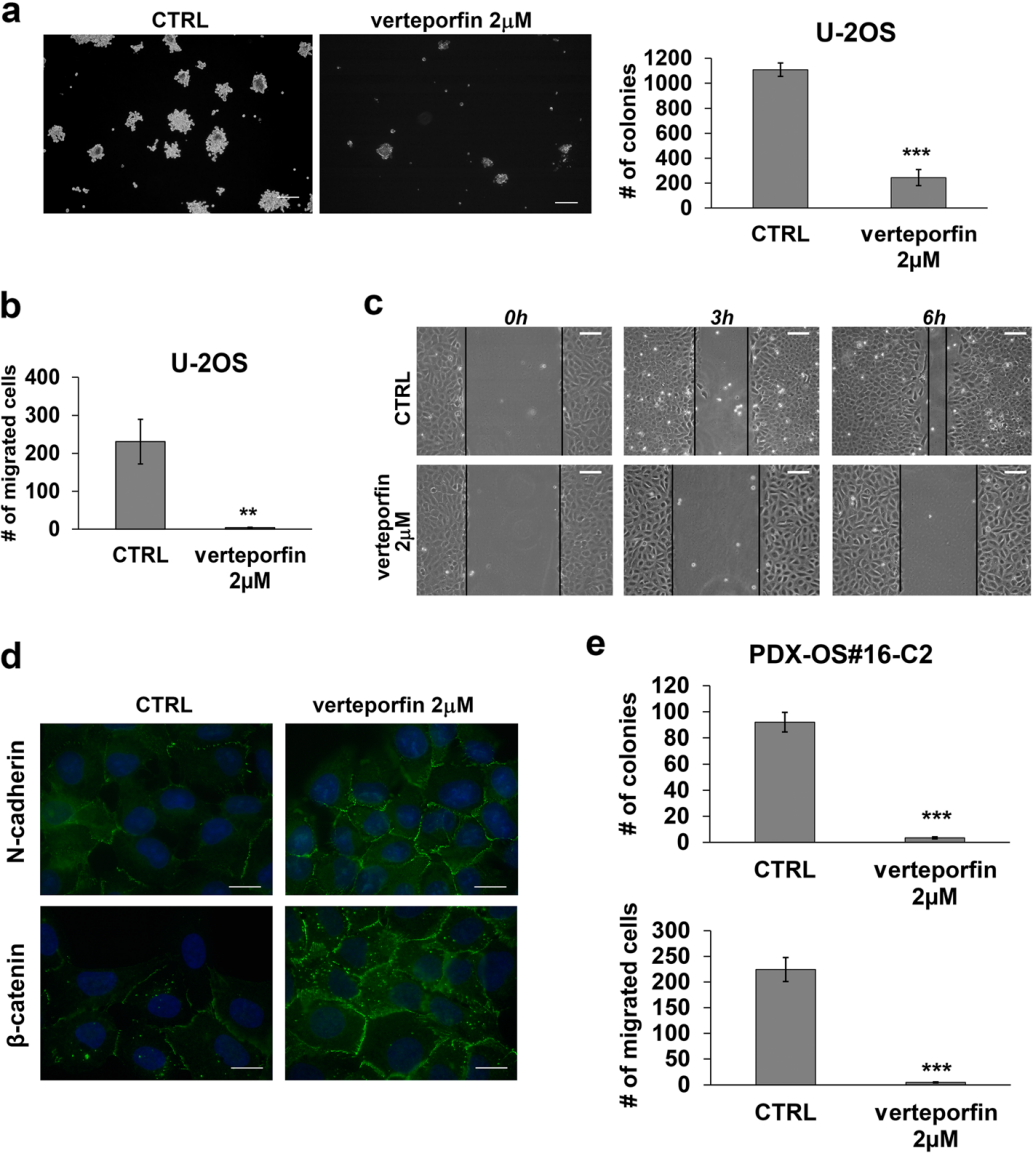
Verteporfin reduces the aggressiveness of OS cells. **a** Effects of verteporfin on the growth of U-2OS cells in anchorage-independent conditions. Each column (right) represents the mean ± SE of three separate experiments. Colonies were counted after 10 days. *** *P* < 0.0001, paired Student’s t test. Representative images (left) of spheroid colonies are shown. Scale bar, 200 μm **b** Effects of verteporfin on the migration of U-2OS cells in Transwell chambers. Each column represents the mean ± SE of three separate experiments. *** *P* < 0.0001, paired Student’s t test. **c** Effects of verteporfin after wound-healing assays scale bar, 100 μm **d** Immunofluorescence staining of N-cadherin and β-catenin in U-2OS cells after 24 h of treatment with verteporfin. Digital images were taken in identical conditions using the image analysis software NIS-Elements; scale bar, 20 μm. **e** Effects of verteporfin on the growth of PDX-OS#16-C2 in anchorage-independent conditions (upper) and on migration of these cells (lower). Each column represents the mean + SE of three separate experiments. *** *P* < 0.0001, paired Student’s t test

To test the therapeutic potential of YAP inhibition in OS, we used verteporfin, a porphyrin compound that was reported to block YAP-TEAD interactions [16]. Verteporfin effectively reduced U-2OS cell viability, with an IC50 value of 1.44 ± 0.46 μM. As demonstrated in other tumors, including synovial sarcoma [25], verteporfin led to a dose- and time-dependent reduction in the expression (Fig. 4a) and activity (Fig. 4b) of YAP. Notably, verteporfin was also able to induce a dose- and time-dependent decrease in ROCK2 expression both at mRNA (Additional file 3: Figure S3) and protein levels (Fig. 4c, d), confirming the functional interconnection between YAP and ROCK2. Verteporfin treatment significantly inhibited the anchorage-independent growth of OS cells (Fig. 5a) and completely abrogated the migration of these cells (Fig. 5b and c). Cells treated with verteporfin showed increased expression and cell-membrane recruitment of N-cadherin and β-catenin (Fig. 5d), thereby displaying the same phenotype that was previously observed after ROCK2 depletion (Additional file 1: Figure S1).

To build on our observations, we confirmed the growth-suppressive effects of verteporfin in three cell lines derived from PDXs, which have been reported to model the genetic features of human tumors, including bone sarcomas, with a high level of fidelity [17, 26–29]. Verteporfin effectively suppressed the cell growth of all three cell lines under standard conditions, with IC50 values ranging from 1 to 2 μM. In addition, in PDX-OS#16-C2, which expressed the highest levels of ROCK2 and YAP (Additional file 4: Figure S4), verteporfin completely suppressed the capability of these cells to form colonies and to migrate (Fig. 5e).

## Discussion

ROCK2 kinase has been described as a critical mediator of biological functions that are implicated in the metastatic processes, including the disruption of adherens junctions, actin cytoskeleton remodeling, the dissociation of cell clusters and increased cell motility [30, 31]. In OS, we have previously shown that ROCK2 is a crucial intracellular mediator of the CD99-induced suppression of cell migration [15]. The inhibition of ROCK2 was shown to impair the migratory and adhesive behavior of OS cells by decreasing the expression of ezrin, an actin-binding protein that leads to cytoskeletal regulation, and by recruiting N-cadherin and β-catenin to the cell membrane. In this study, we expanded these observations and showed that when ROCK2 expression was stably downregulated in OS cells, tumor growth was significantly inhibited in NSG mice and, notably, tumors completely lost the capability to disseminate and to form spontaneous metastases in the lungs. These results strongly support the idea of specifically targeting ROCK2 kinase to prevent the formation of metastasis in OS. Although neoadjuvant chemotherapy has substantially improved the outcome of patients with localized disease, very few, if any, novel drugs are available for patients who fail to respond to first-line treatments or who have metastasis at diagnosis [32]. Our preclinical data indicate a potential therapeutic use for ROCK2 inhibitors. However, despite the interest from pharmaceutical companies in the ROCK pathway, only a few ROCK inhibitors have reached either clinical trials or the market [33]. In fact, only fasudil and ripasudil have been approved for clinical use to treat cerebral vasospasm and glaucoma [34, 35].

The potential safety concerns related to the use of ROCK inhibitors, together with the fact that the currently developed compounds have shown only moderate kinase selectivity (either against the two isoforms ROCK1 and ROCK2 or to a number of other kinases), have limited their use as systemic therapies in cancer clinical trials. Studying the downstream effectors of ROCK2 in the appropriate cellular context may thus allow the direct inhibition of this kinase to be bypassed, leading to the identification of alternative therapeutic approaches. In recent years, the connection between ROCK signaling and YAP activity in the context of cellular mechanoresponses has emerged. In particular, ROCK was found to be involved in the maintenance of the nuclear localization of YAP, thereby enhancing the activity of YAP [11, 14]. In accordance with these results, we demonstrated that ROCK2 also promotes YAP activity in OS. In fact, in the tumors that developed in mice, the deprivation of ROCK2 occurred in parallel with the reduction in YAP expression and its transcriptional activity. In vitro, the inhibition of ROCK2 activity, either by pharmacological inhibition or silencing, induced a dose- and time-dependent reduction in the expression of YAP and its downstream target genes *CTGF*, *CYR61* and *CCND1*, confirming the functional connection between these two intracellular mediators. YAP overexpression has been observed in several tumors, and high YAP expression levels have been correlated with poor patient prognosis in ovarian, non-small cell lung cancer and esophageal squamous cell carcinoma [36–38]. In OS, YAP is expressed in a vast majority of tumor samples [39]. However, only the presence of YAP in the nucleus, which is related to its transcriptional activity, but not the expression of ROCK2 or the general expression of YAP, was found to be associated with a higher probability of patient relapse. Accordingly, the incidence of metastasis was higher in patients that expressed YAP in the nuclei of tumor cells, and worse patient prognosis was associated with the level of YAP activity. In fact, the patients with the highest expression of YAP in the nucleus had a worse prognosis, either in terms of RFS or OVS, and died with a higher frequency than other patients. Therefore, these clinical data support the therapeutic potential of targeting YAP. Liu-Chittenden et al*.* [16] found that three compounds, which were related to porphyrin, out of > 3300 drugs inhibited the transcriptional activity of YAP. One of these compounds, verteporfin, is clinically used as a photosensitizer in photocoagulation therapy for macular degeneration [40]. More recently, verteporfin was shown to be effective at blocking the assembly of the functional YAP-TEAD transcription factor [16, 41, 42], suggesting the application of this compound as an anticancer agent. In OS, we showed that verteporfin, in addition to downregulating the expression and activity of YAP, significantly impaired tumor cell growth, either in standard and anchorage-independent conditions, and completely disrupted cell migration. YAP likely exerts its control on migration, at least in part, through the transcriptional regulation of the *CTGF* and *CYR61* promoters. These genes, which belong to the CCN (Cyr61, CTGF and Nephroblastoma overexpressed gene) family [43], promote the epithelial-mesenchymal transition (EMT) process, allowing cancer cells to migrate and to disseminate to distant organs [44–46]. The overexpression of Cyr61 (CCN1) in the low-metastatic variant of the human SaOS-2 OS cell line increased cell proliferation and promoted lung metastasis [47], and both Cyr61 and CTGF (CCN2) have been implicated in the progression of bone metastases in other cancers [48]. Moreover, both Cyr61 and CTGF were shown to play a pivotal role in osteogenesis, and their expression decreased during the differentiation of OS cells to osteoblasts [49]. Therefore, the modulation of these genes by YAP may have a large impact on the progression of tumors that grow in the bone microenvironment, such as primary bone tumors and bone metastases. In addition to reducing the expression of *CTGF* and *CYR61*, our findings indicate that verteporfin also affects the ROCK2 protein, increases the expression and recruitment of N-cadherin and β-catenin to the cell membrane. A positive-feedback mechanism between YAP and ROCK2 has been recently demonstrated by Sugimoto et al. [50], who showed that in response to extracellular matrix rigidity, ROCK2 enhances the activation of YAP, and YAP, in turn, induces ROCK2 expression by directly activating the *ROCK2* promoter. Our results are in line with these findings and suggest that targeting YAP could be a rational strategy to inhibit multiple effects of the ROCK2/YAP axis that affect the invasive phenotype of tumor cells. Interestingly, YAP also plays important roles in immune cells and is involved in drug resistance [51–53], further supporting the systemic use of YAP inhibitors, such as verteporfin, as adjuvant agents to potentiate chemotherapy. Although YAP-independent effects have also been described for verteporfin, supporting the view that this compound is a multitarget drug that interacts with several proteins involved in major cellular processes, this apparent lack of specificity does not preclude its possible clinical use. This drug still has the advantage of being an FDA-approved photodynamic therapy, and for rare tumors, such as OS, this approval could make a difference.

Some efficacy of agents like pazopanib, which were reported to inhibit multiple targets including YAP [54], have been reported on case reports [55, 56], further supporting the investment in this area of research.

## Conclusion

Very few, if any, effective treatment options exist for OS patients with metastatic disease. Thus, we desperately need to identify the pathways that promote metastasis and to determine how these pathways act in this specific cellular context. This paper suggests that ROCK2 is an important driver of OS migration and metastasis and provides evidence that the dysregulation of ROCK2 sustains YAP activity. Patients with the nuclear expression of YAP have a worse prognosis due to a higher incidence of metastasis and may benefit from drugs, such as verteporfin, that inhibit YAP activity. We showed that this agent inhibits YAP transcriptional activity and decreases ROCK2 expression, thus activating a positive-feedback loop that remarkably impacts OS growth and dissemination.

## Supplementary information

**Additional file 1: Figure S1.** (a) Evaluation of expression and intracellular localization of ROCK2, N-cadherin and β-catenin in ROCK2 depleted U-2OS cell variants by immunoflourescence. Digital images were taken in identical conditions using the Image Analysis Software Nis Elements. Magnification × 600, scale bar 20 µm (b) Western blotting of ROCK2 in the same cellular variants to confirm ROCK2 depletion. GAPDH was used as a loading control. (c) Anchorage-independent growth of controls and ROCK2 depleted cells. Each column represents mean ± SE of at least two separate experiments. ***p < 0.0001, paired Student’s t-test; (d) Migration ability of controls and ROCK2 depleted cells. Each column represents mean ± SE of three separate experiments. * *p* < 0.05; ** *p* < 0.01, paired Student’s t-test.

**Additional file 2: Figure S2.** ROCK2 and YAP expression evaluated by the immunostaining of paraffin-embedded tissue sample. Representative images from another tumor developed after injection of control (U-2/SCR) or silenced cells (U-2/shROCK2#78). Scale bar 50 μm; 200x of magnification with zoomed insert to show details.

**Additional file 3: Figure S3.** Dose- and time-dependent qPCR analysis of the expression of ROCK2 in U-2OS parental cells. Data indicate the percentage of ROCK2 mRNA inhibition with respect to control. Data are shown as mean +/- SE of three separate experiments (* *p* < 0.05, ** *p* < 0.01, *** *p* < 0.001, Student's t test). GAPDH was used as a housekeeping gene.

**Additional file 4: Figure S4.** Western blotting of ROCK2 and YAP in PDX derived cell lines. Equal loading was monitored by anti-GAPDH blotting.

## Data Availability

Further information and requests for resources and reagents should be directed to and will be fulfilled by the Lead Contact, Katia Scotlandi (katia.scotlandi@ior.it).

## Abbreviations

*CCND1*: Cyclin D1
*CTGF*: Connective Tissue Growth Factor
*CYR61*: Cysteine Rich Angiogenic Inducer 61
DMSO: Dimetilsulfoxide
FBS: Fetal bovine serum
*GAPDH*: Glyceraldehyde-3-phosphate dehydrogenase
IHC: Immunohistochemistry
IMDM: Iscove’s modified Dulbecco’s medium
NA: Not available
NED: No evidence of disease
NSG: NOD Scid gamma
OS: Osteosarcoma
OVS: Overall survival
PDX: Patient-derived xenograft
q-PCR: Quantitative real time polymerase chain reaction
REL: Relapsed
RFS: Relapse-free survival
ROCK1: Rho associated coiled-coil containing protein kinases 1
ROCK2: Rho associated coiled-coil containing protein kinases 2
YAP: Yes associated protein 1
