# Supplementary figures and images for: ROCK2 deprivation leads to the inhibition of tumor growth and metastatic potential in osteosarcoma cells through the modulation of YAP activity
Source: J Exp Clin Cancer Res. 2019 Dec 26;38:503. doi: 10.1186/s13046-019-1506-3 (PMC6933701; doi:10.1186/s13046-019-1506-3)

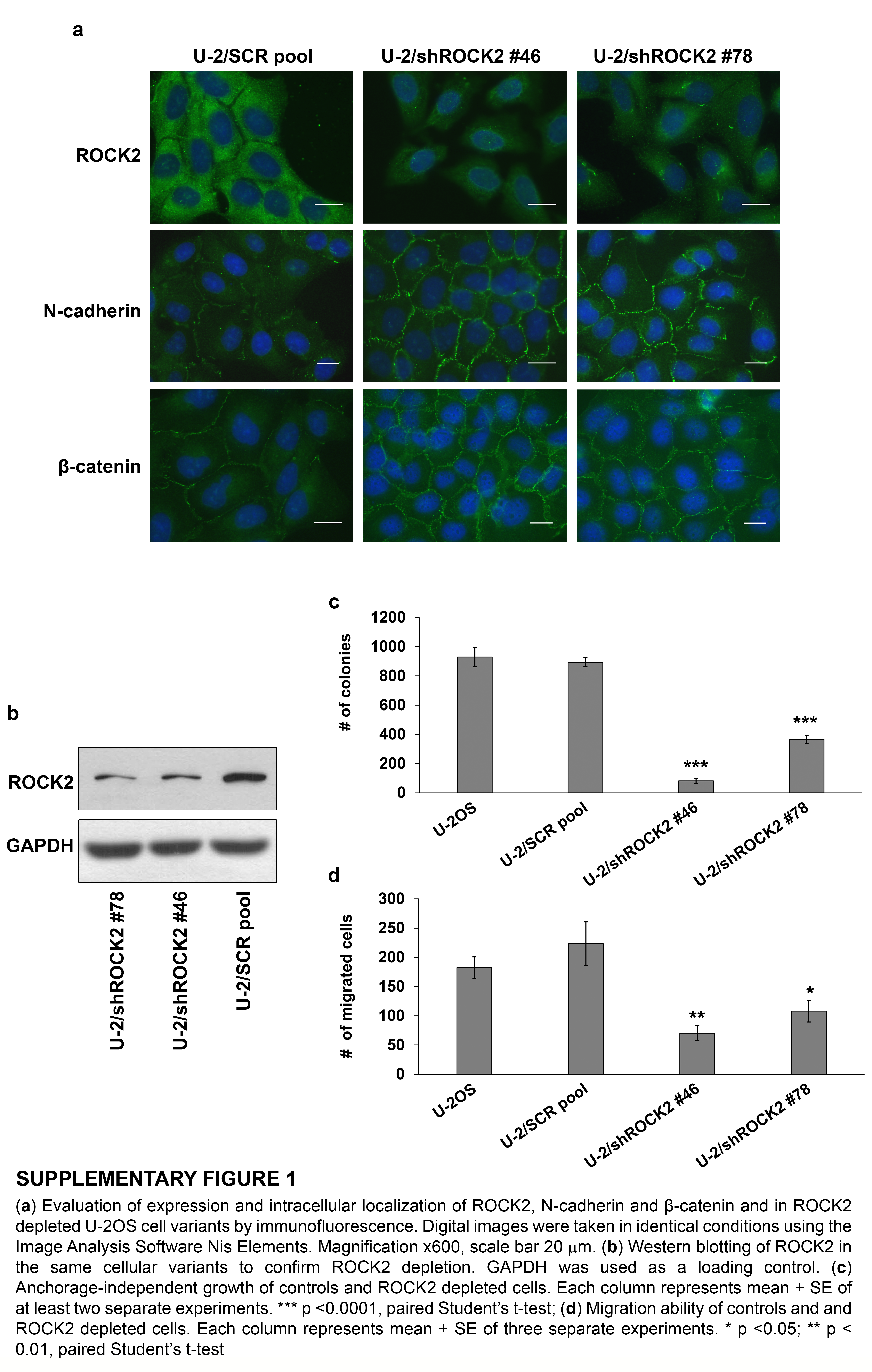

Supplement: Supplementary file 1 — Additional file 1: Figure S1. (a) Evaluation of expression and intracellular localization of ROCK2, N-cadherin and β-catenin in ROCK2 depleted U-2OS cell variants by immunoflourescence. Digital images were taken in identical conditions using the Image Analysis Software Nis Elements. Magnification × 600, scale bar 20 µm (b) Western blotting of ROCK2 in the same cellular variants to confirm ROCK2 depletion. GAPDH was used as a loading control. (c) Anchorage-independent growth of controls and ROCK2 depleted cells. Each column represents mean ± SE of at least two separate experiments. ***p < 0.0001, paired Student’s t-test; (d) Migration ability of controls and ROCK2 depleted cells. Each column represents mean ± SE of three separate experiments. * p < 0.05; ** p < 0.01, paired Student’s t-test. [file 13046_2019_1506_MOESM1_ESM.tif]

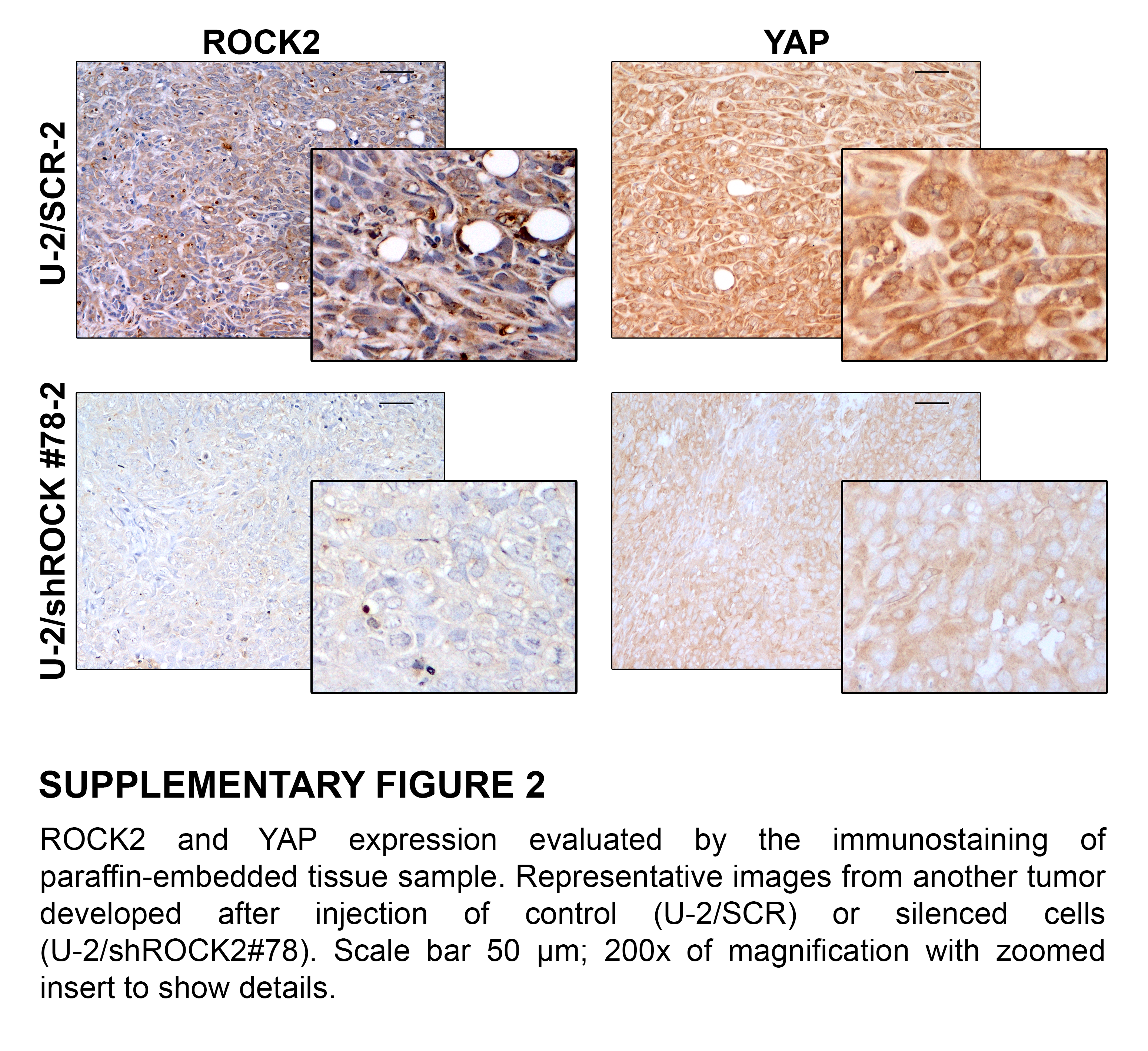

Supplement: Supplementary file 2 — Additional file 2: Figure S2. ROCK2 and YAP expression evaluated by the immunostaining of paraffin-embedded tissue sample. Representative images from another tumor developed after injection of control (U-2/SCR) or silenced cells (U-2/shROCK2#78). Scale bar 50 μm; 200x of magnification with zoomed insert to show details. [file 13046_2019_1506_MOESM2_ESM.tif]

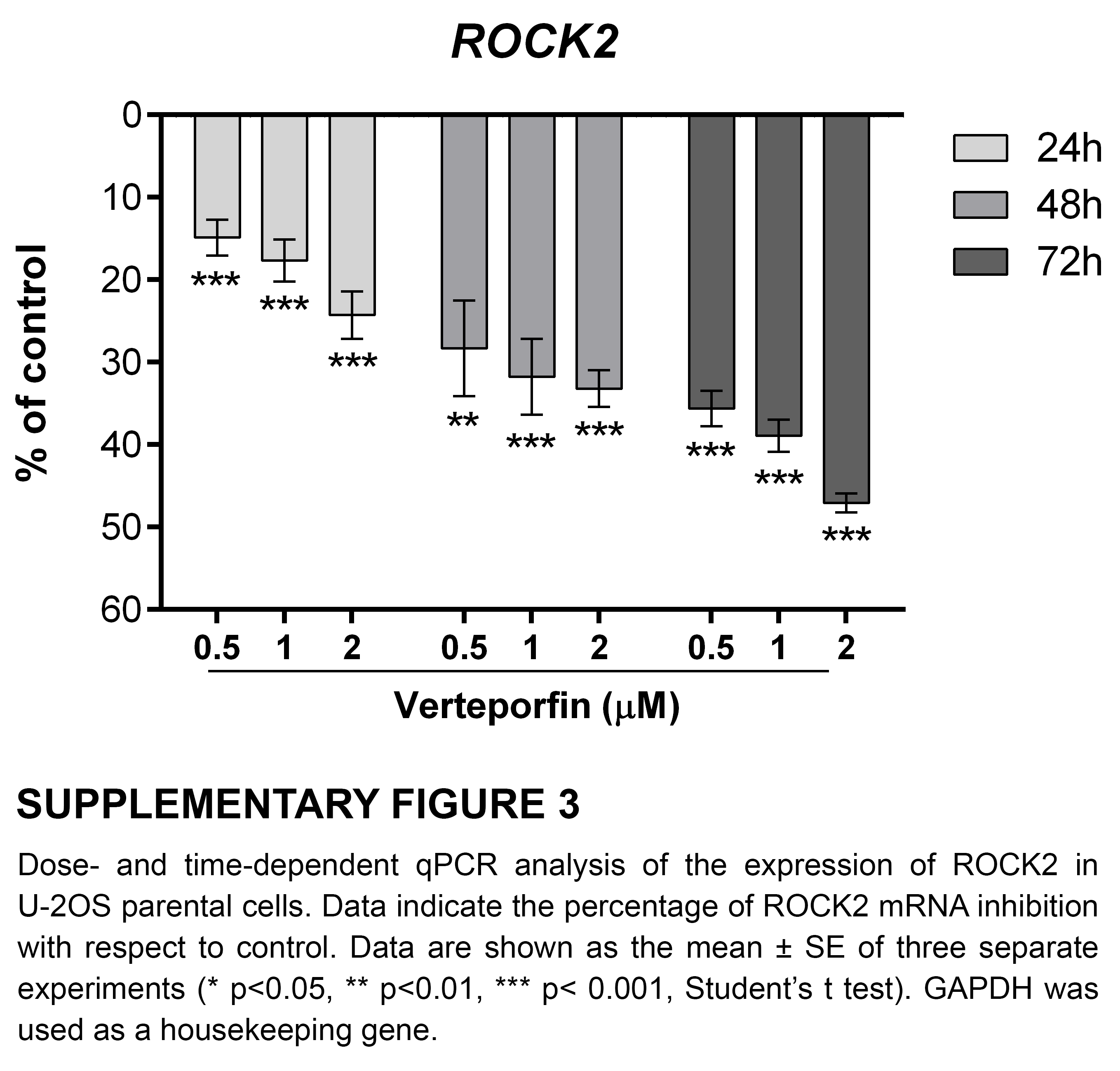

Supplement: Supplementary file 3 — Additional file 3: Figure S3. Dose- and time-dependent qPCR analysis of the expression of ROCK2 in U-2OS parental cells. Data indicate the percentage of ROCK2 mRNA inhibition with respect to control. Data are shown as mean +/- SE of three separate experiments (* p < 0.05, ** p < 0.01, *** p < 0.001, Student's t test). GAPDH was used as a housekeeping gene. [file 13046_2019_1506_MOESM3_ESM.tif]

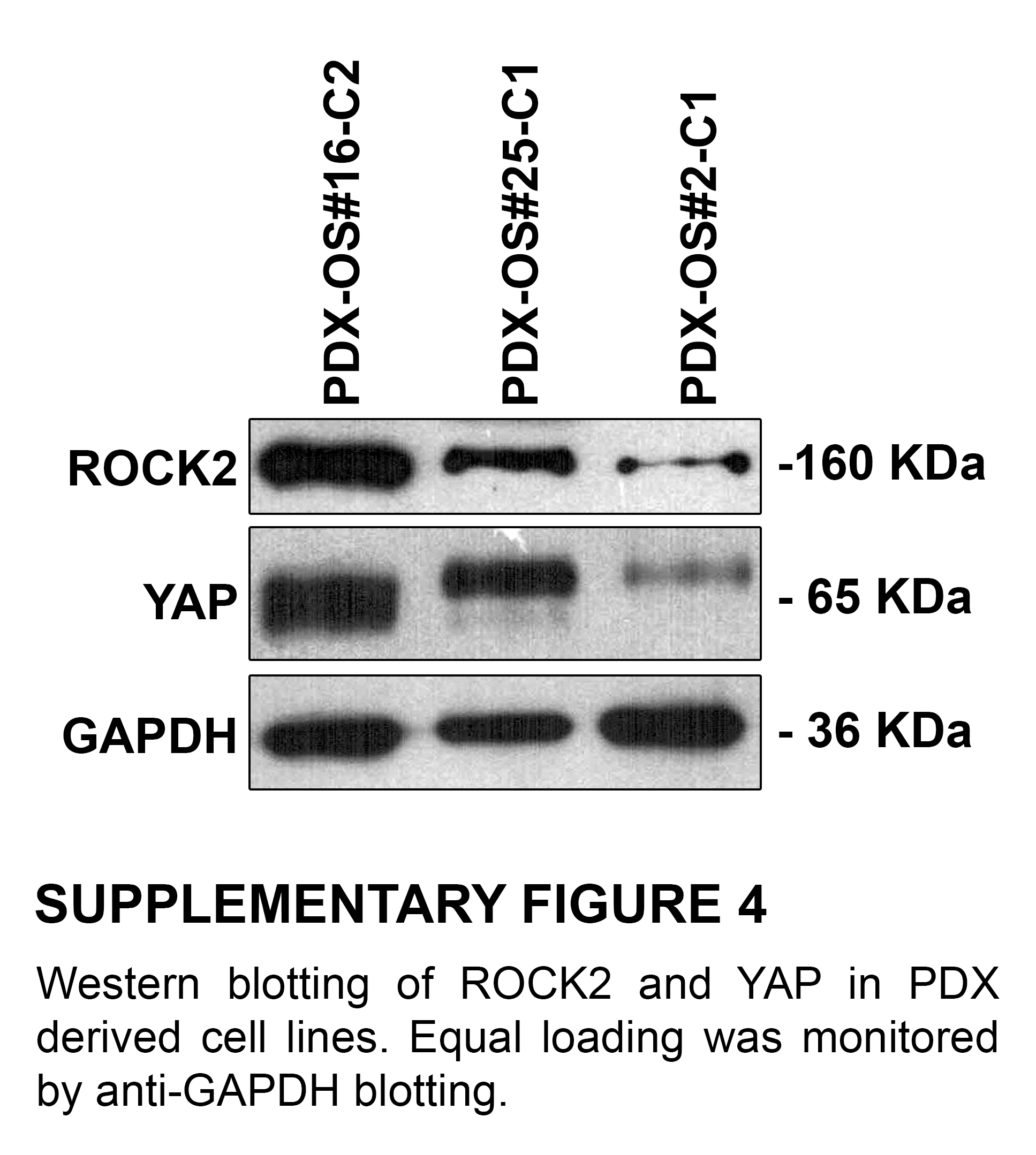

Supplement: Supplementary file 4 — Additional file 4: Figure S4. Western blotting of ROCK2 and YAP in PDX derived cell lines. Equal loading was monitored by anti-GAPDH blotting. [file 13046_2019_1506_MOESM4_ESM.tif]
